# Silent progression: cardiac amyloidosis unmasking IgG lambda myeloma in an elderly patient

**DOI:** 10.1186/s43044-026-00715-w

**Published:** 2026-01-06

**Authors:** Soufiane Touiti, Meriem Bouali, Loubna El Bahri, Iliyasse Asfalou, Zouhair Lakhal, Aatif Benyass

**Affiliations:** 1 Cardiac Catheterization Unit, Military Hospital Mohamed V, Rabat, Morocco; 2 Non invasive cardiac explorations , Military Hospital Mohamed V, Rabat, Morocco

**Keywords:** Cardiac amyloidosis, AL amyloidosis, Multiple myeloma, Echocardiography, Heart failure with preserved ejection fraction

## Abstract

**Background:**

Cardiac amyloidosis is an underrecognized etiology of heart failure with preserved ejection fraction (HFpEF), particularly in elderly patients. Light-chain (AL) amyloidosis, when associated with multiple myeloma, is highly aggressive and portends a poor prognosis, especially in advanced cardiac stages.

**Case presentation:**

We report the case of a 79-year-old male with type 2 diabetes, hypertension, and a history of pacemaker implantation for complete atrioventricular block in the context of atrial fibrillation. He was admitted with progressive exertional dyspnea and an episode of syncope. Clinical examination revealed fine basal crackles and signs of decompensated heart failure. ECG demonstrated a paced rhythm. Echocardiography and cardiac MRI revealed concentric left ventricular hypertrophy with a sparkling myocardial texture, biatrial dilation, restrictive filling pattern, and diffuse subendocardial late gadolinium enhancement—features highly suggestive of cardiac amyloidosis. Laboratory tests revealed anemia, nephrotic syndrome, elevated troponin and NT-proBNP, and a monoclonal IgG lambda spike. Renal biopsy demonstrated amyloid deposits with Congo red positivity and light-chain (lambda) restriction, confirming the diagnosis of AL amyloidosis. Bone marrow biopsy confirmed the diagnosis of multiple myeloma with plasma cell infiltration. Based on clinical and laboratory findings, the patient was classified as Mayo stage IIIB AL cardiac amyloidosis and ISS stage I multiple myeloma. He received bortezomib-cyclophosphamide-based chemotherapy and supportive care, but unfortunately died five months after diagnosis.

**Discussion:**

This case highlights the importance of early recognition of cardiac amyloidosis in elderly patients with unexplained heart failure and monoclonal gammopathy. Echocardiography plays a pivotal role in early disease suspicion, particularly in resource-limited settings, while cardiac MRI serves as a complementary tool for assessing myocardial involvement. Despite advances in treatment, outcomes remain poor in advanced cardiac involvement. Early intervention may improve prognosis, underscoring the need for heightened clinical awareness.

**Conclusion:**

Infiltrative cardiomyopathies like AL amyloidosis should be considered in elderly patients with heart failure and systemic red flags. Timely diagnosis and multidisciplinary management are essential but often insufficient in advanced stages.

## Introduction

Cardiac amyloidosis is an infiltrative cardiomyopathy caused by extracellular deposition of misfolded protein fibrils within the myocardium, leading to progressive diastolic dysfunction and restrictive physiology. Among its various forms, immunoglobulin light-chain (AL) amyloidosis is particularly aggressive, often associated with rapid deterioration when cardiac involvement occurs. More than 50% of patients with AL amyloidosis develop cardiac involvement, which is the main determinant of prognosis—median survival is often limited to a few months without treatment or when diagnosis is delayed [1, 2].

The clinical presentation of cardiac amyloidosis is frequently nonspecific, often mimicking hypertensive heart disease or heart failure with preserved ejection fraction (HFpEF), especially in older adults [3]. AL amyloidosis is typically associated with an underlying plasma cell dyscrasia, such as multiple myeloma or monoclonal gammopathy of undetermined significance (MGUS) [4]. In some cases, cardiac symptoms may precede hematologic signs, delaying diagnosis and treatment.

We report a case of advanced AL cardiac amyloidosis revealing underlying IgG lambda multiple myeloma in a 79-year-old man. This case highlights the diagnostic and therapeutic challenges inherent to this often-overlooked condition.

## Case report

A 79-year-old man with a medical history of type 2 diabetes mellitus, hypertension, and permanent atrial fibrillation with dual-chamber pacemaker implantation in 2022 for complete atrioventricular block, presented with progressive exertional dyspnea over three months. His symptoms progressed from NYHA class II to class III, and he experienced a brief syncope (~ 10 s) while climbing stairs, without post-ictal confusion or focal neurological deficit. This, along with a global decline in functional status, prompted hospital admission.

On admission, he was alert and oriented. Respiratory rate was 23 breaths/min, heart rate was 65 bpm (regular), and blood pressure was 110/50 mmHg. Cardiac auscultation revealed regular heart sounds without murmurs or added sounds. Fine bibasilar crackles were noted on pulmonary examination; the remainder of the exam was unremarkable.

Chest radiography was normal. Electrocardiogram (ECG) showed a ventricular-paced rhythm with a controlled rate of 65 bpm, without conduction or repolarization abnormalities.

**Fig. 1:**
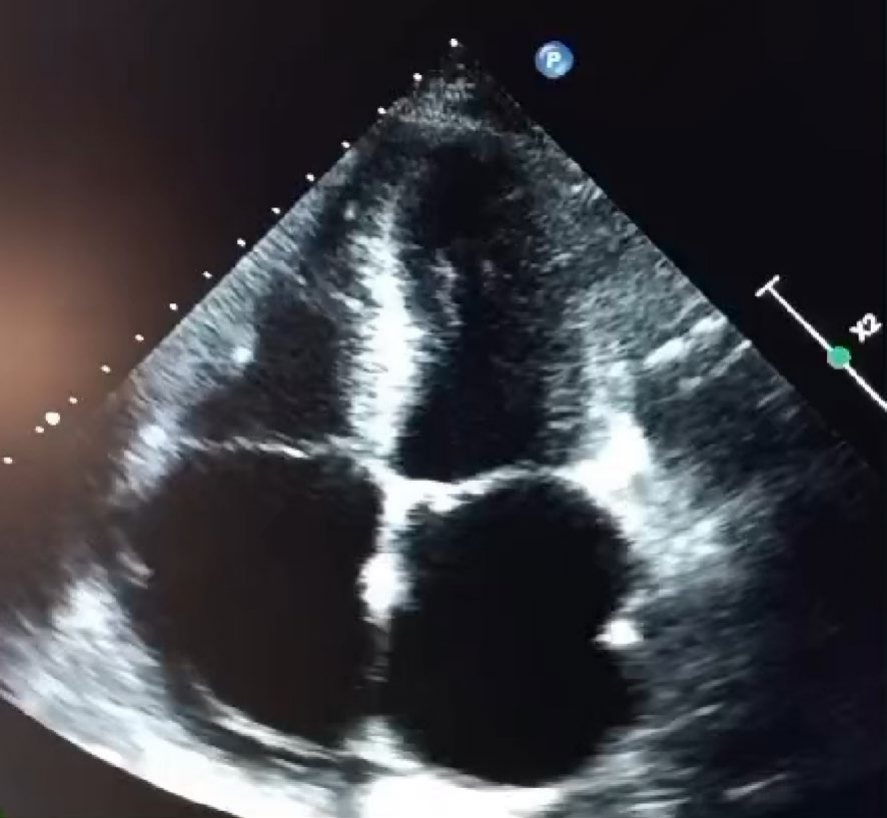
Apical four-chamber view showing concentric LV hypertrophy with granular myocardial texture and biatrial enlargement

**Fig. 2:**
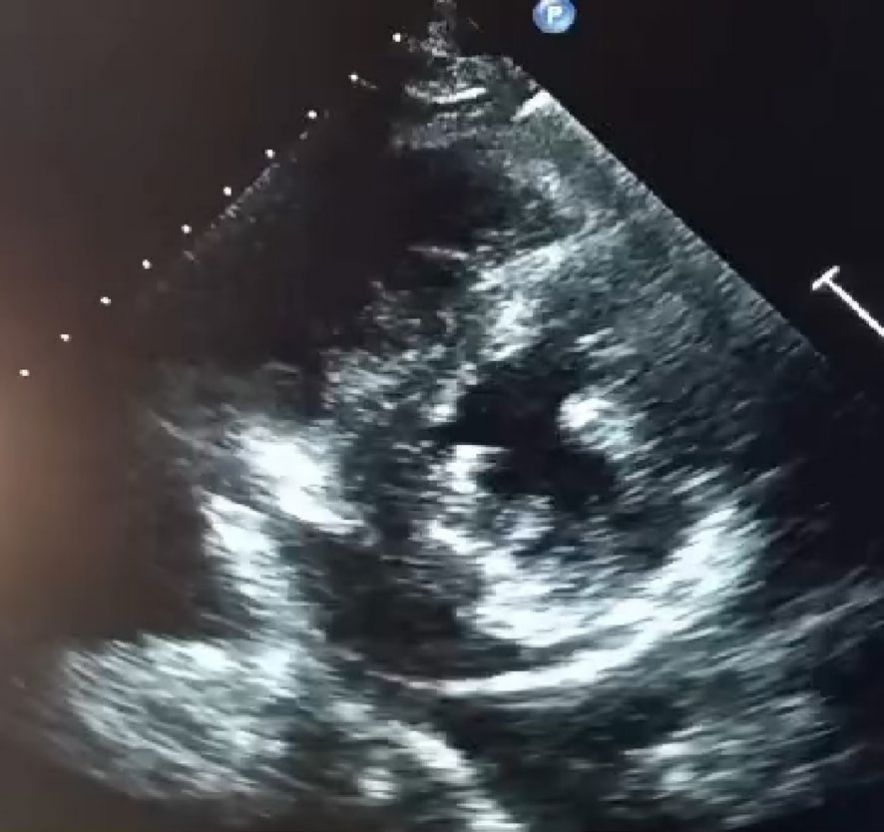
Parasternal short-axis view confirming concentric LV thickening

**Fig. 3:**
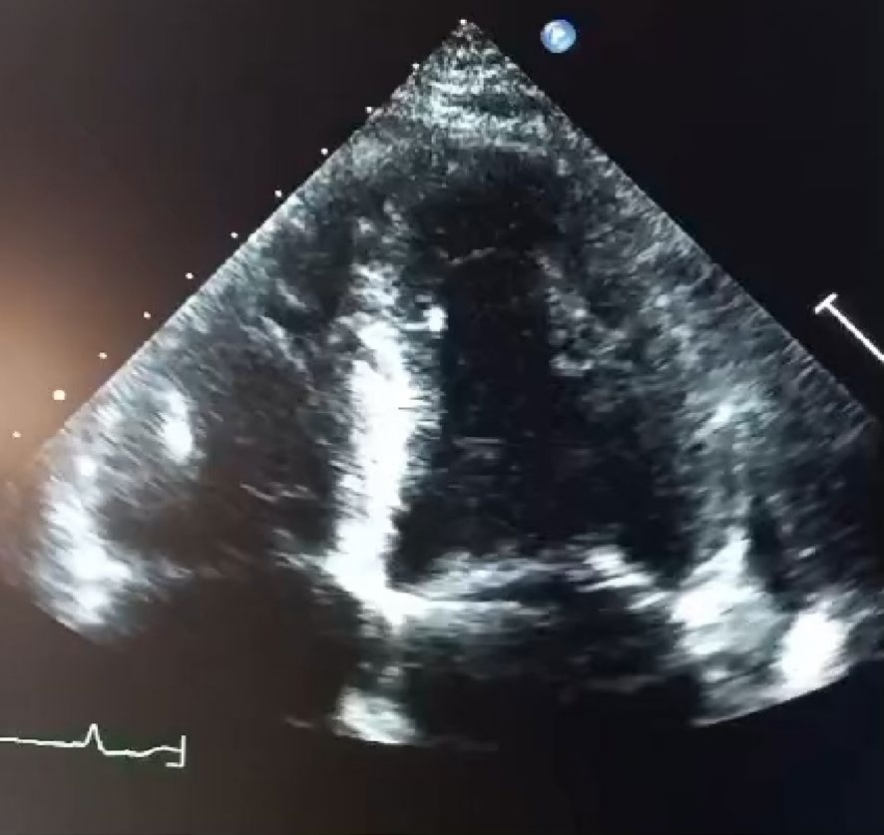
Apical four-chamber view with similar findings

**Fig. 4:**
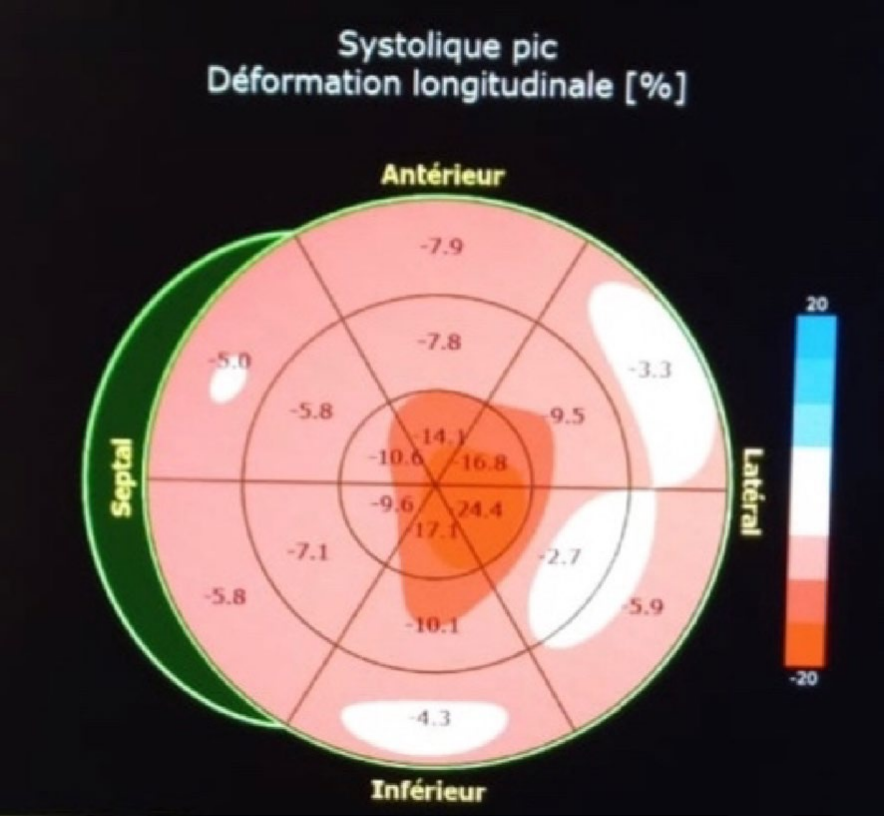
Bull’s-eye plot showing severely reduced global longitudinal strain with apical sparing—typical “cherry-on-top” pattern indicative of cardiac amyloidosis

Transthoracic echocardiography (TTE) (Figs. 1, 2, 3 and 4) revealed a non-dilated left ventricle with preserved systolic function (left ventricular ejection fraction estimated at 58%) with concentric hypertrophy and a granular or “sparkling” myocardial appearance, highly suggestive of an infiltrative cardiomyopathy. Both the interventricular septum and the posterior wall were thickened, measuring 14 mm. Biatrial enlargement was noted. Doppler evaluation demonstrated a restrictive mitral inflow pattern, consistent with elevated left ventricular filling pressures. Speckle-tracking analysis showed a significantly reduced global longitudinal strain, with relative apical sparing—producing the classic “cherry-on-top” pattern on the bull’s-eye plot, strongly indicative of cardiac amyloidosis.

**Fig. 5:**
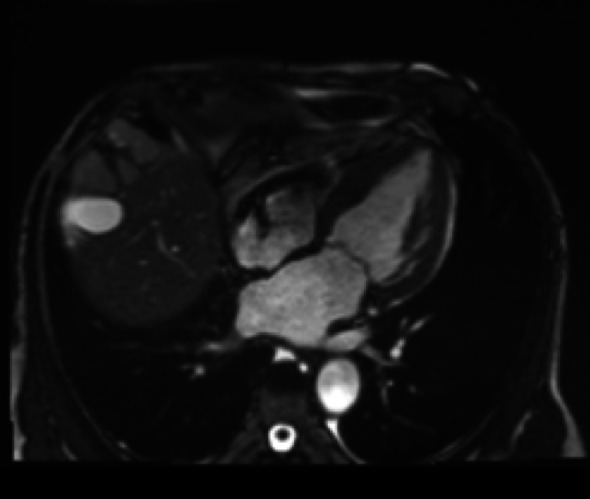
A Axial cine SSFP view showing concentric left ventricular hypertrophy

**Fig. 6:**
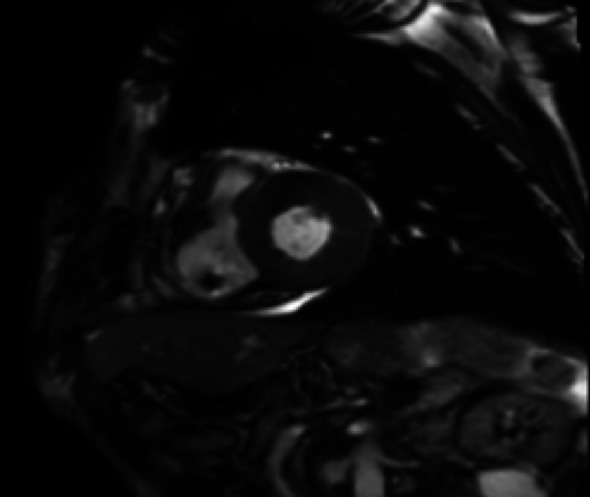
Short-axis T2-weighted image showing thickened myocardium without edema

**Fig. 7:**
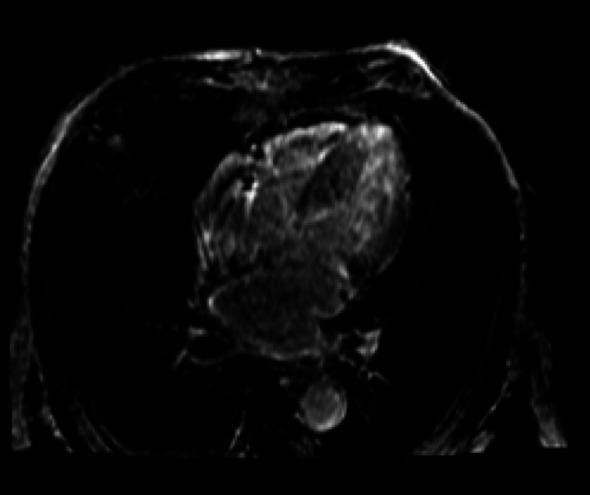
Four-chamber LGE view demonstrating diffuse subendocardial enhancement of both ventricles, consistent with advanced cardiac amyloid infiltration

Cardiac magnetic resonance imaging (CMR) (Figs. 5, 6 and 7) was performed as a complementary diagnostic tool. It demonstrated marked concentric left ventricular hypertrophy with diffuse subendocardial late gadolinium enhancement (LGE), notably involving the interventricular septum and extending to all myocardial walls. Native T1 mapping revealed significantly elevated values (1310 ms in the lateral wall), and the calculated extracellular volume (ECV) was 38%—both highly consistent with diffuse myocardial amyloid infiltration.

Laboratory evaluation showed normocytic normochromic anemia (Hb 11.8 g/dL), chronic kidney disease (eGFR 49 mL/min/1.73 m²), and nephrotic syndrome, with proteinuria (urine protein-to-creatinine ratio: 158 mg/mmol; albumin-to-creatinine ratio: 21 mg/mmol), and low serum albumin (13 g/L). Total 24-hour proteinuria was 29 g.

Cardiac biomarkers were significantly elevated: troponin I at 132 ng/L (~ 3x upper limit of normal) and NT-proBNP at 8828 pg/mL.

Serum protein electrophoresis (SPEP) showed a monoclonal spike in the gamma region (20.5 g/L). Serum immunofixation identified an IgG lambda monoclonal protein. Serum free light chain assay showed markedly elevated lambda light chains (120 mg/L), normal kappa light chains (28 mg/L), resulting in an abnormal kappa/lambda ratio of 0.23. Bence-Jones proteinuria was negative.

Bone marrow aspiration revealed 10–12% dystrophic plasma cells. Immunohistochemical staining confirmed plasma cell infiltration expressing CD56, consistent with multiple myeloma. In addition, histological examination of renal demonstrated amyloid deposits with Congo red positivity and light-chain (lambda) restriction, confirming the diagnosis of AL amyloidosis.

A skeletal survey, including skull X-rays and spinal/pelvic MRI, showed no lytic lesions. Serum calcium was normal (93 mg/dL). The International Staging System (ISS) score was 1 (β2-microglobulin 3.12 mg/L; serum albumin 13 g/L). Cardiac amyloidosis was staged as Mayo Clinic stage IIIB.

The patient’s heart failure was managed with intravenous loop diuretics. Anticoagulation for atrial fibrillation was maintained. After multidisciplinary evaluation, chemotherapy with bortezomib and cyclophosphamide (VC protocol) was initiated. Doxycycline was used as adjunctive therapy for its potential pleiotropic effects on amyloid fibril stabilization, and antiviral prophylaxis with valaciclovir was prescribed.

Despite optimal treatment, the patient’s condition progressively worsened, and he passed away five months after diagnosis.

## Discussion

Cardiac amyloidosis, particularly light-chain (AL) amyloidosis, represents a critical diagnostic and prognostic inflection point in patients presenting with heart failure with preserved ejection fraction (HFpEF). The disease results from the extracellular deposition of misfolded immunoglobulin light chains produced by clonal plasma cells, most commonly in the myocardium and kidneys, but often with systemic involvement [1]. Cardiac amyloidosis may mimic or coexist with more common cardiovascular conditions, leading to frequent under-recognition and delayed treatment — delays that are often fatal [5].

In this case, the patient’s presentation with progressive dyspnea, syncope, and signs of HFpEF in the context of preserved left ventricular ejection fraction (LVEF) but increased myocardial thickness was initially non-specific. However, several “red flag” findings [6] pointed toward an infiltrative process including unexplained LV hypertrophy, nephrotic syndrome, elevated cardiac biomarkers, and monoclonal gammopathy—pointed toward AL amyloidosis.

Importantly, echocardiography remains the cornerstone of early suspicion for cardiac amyloidosis. It is widely available, non-invasive, rapid, and particularly valuable in resource-limited settings where advanced imaging may not be readily accessible. Typical echocardiographic features include concentric ventricular thickening with small cavity size, biatrial enlargement, restrictive filling patterns, and reduced global longitudinal strain with relative apical sparing. In many cases, echocardiography provides the first and most crucial clue, enabling early referral for confirmatory testing and hematologic evaluation [6]. 

In elderly patients, cardiac amyloidosis is an increasingly recognized cause of HFpEF. In fact, a 2019 autopsy series found that up to 25% of patients over 80 years with HFpEF had evidence of cardiac transthyretin amyloidosis (ATTR) [2]. While AL amyloidosis is less prevalent than ATTR in this age group, it carries a far worse prognosis, especially in the presence of cardiac involvement [7].

In clinical practice, AL amyloidosis should be suspected in any patient with unexplained heart failure, especially when accompanied by systemic signs such as macroglossia, periorbital purpura, weight loss, syncope, or nephrotic syndrome [8]. Notably, in our patient, the history of conduction system disease requiring pacemaker implantation—in the absence of significant ischemia—should have raised earlier suspicion for amyloid infiltration.

Cardiac magnetic resonance (CMR) is a highly sensitive tool for detecting myocardial infiltration, typically demonstrating diffuse subendocardial or transmural late gadolinium enhancement, along with increased native T1 values and extracellular volume (ECV) [9, 10]. In this case, the patient’s MRI showed diffuse LGE and an ECV of 38%, consistent with severe myocardial amyloid infiltration. Elevated troponin and NT-proBNP are also strongly prognostic. The Mayo Clinic 2012 staging system incorporates these markers, and stage IIIB disease, as in this case, carries a median survival of only 3–4 months, even with therapy [11].

Although AL amyloidosis is associated with underlying plasma cell dyscrasias, only ~ 10–15% of patients with multiple myeloma develop systemic AL amyloidosis [12]. Conversely, AL amyloidosis can precede or co-exist with multiple myeloma in up to 30% of cases [13]. This patient had IgG lambda multiple myeloma with moderate plasma cell infiltration (10–12%), fitting the diagnostic criteria for both disorders. The lack of bone lesions or hypercalcemia, coupled with renal and cardiac dysfunction, pointed toward amyloid organ damage as the primary manifestation.

The prognostic scoring in multiple myeloma (ISS stage I in this case) does not capture the impact of cardiac amyloid, which often independently dictates outcome. This dichotomy between hematologic and cardiac severity complicates treatment planning [14]. 

Multidisciplinary collaboration is key. Treatment strategies must balance the aggressive nature of the plasma cell dyscrasia with the fragility imposed by cardiac dysfunction [15]. In this patient, despite the appropriate chemotherapy and supportive management, disease progression was relentless due to the advanced cardiac amyloid infiltration.

The mainstay of AL amyloidosis therapy is eliminating the amyloidogenic plasma cell clone, typically using bortezomib-based regimens [14]. In this case, the patient was started on a bortezomib-cyclophosphamide regimen (VC protocol) with antimicrobial prophylaxis. Unfortunately, advanced cardiac involvement limits chemotherapy tolerability, and even well-selected patients with stage IIIB disease may not survive beyond a few months [11].

Emerging therapies, such as daratumumab (anti-CD38 monoclonal antibody), have shown promise in AL amyloidosis when added to standard regimens (e.g., Dara-VCD), improving hematologic response and survival [16]. However, such therapies may not be accessible in certain healthcare settings or appropriate in elderly frail patients with multiorgan failure.

This case poignantly illustrates the crucial role of early diagnosis. Had the diagnosis of amyloidosis been considered earlier—perhaps at the time of pacemaker implantation for idiopathic AV block—the patient might have received therapy before reaching irreversible cardiac decompensation.

## Conclusion

Cardiac amyloidosis should be considered in elderly patients with unexplained HFpEF, conduction disturbances, or disproportionate LV hypertrophy. The presence of red flags such as proteinuria, low serum albumin, and monoclonal gammopathy should prompt thorough diagnostic workup. This case illustrates how cardiac involvement can serve as the initial manifestation of underlying multiple myeloma and highlights the severe prognosis associated with late-stage AL amyloidosis. Early diagnosis, multidisciplinary management, and access to novel therapies are key to improving survival in these patients.

## Data Availability

No datasets were generated or analysed during the current study.

## Abbreviations

VC protocol: Bortezomib–cyclophosphamide protocol
TTE: Transthoracic echocardiography
SSFP: Steady-state free precession
SPEP: Serum protein electrophoresis
NYHA: New York Heart Association
NT-proBNP: N-terminal pro–B-type natriuretic peptide
MRI: Magnetic resonance imaging
MGUS: Monoclonal gammopathy of undetermined significance
LVEF: Left ventricular ejection fraction
LV: Left ventricle / ventricular
LGE: Late gadolinium enhancement
ISS: International Staging System
HFpEF: Heart failure with preserved ejection fraction
GLS: Global longitudinal strain
ESC: European Society of Cardiology
EF: Ejection fraction
ECV: Extracellular volume
ECG: Electrocardiogram
DARA-VCD: Daratumumab–bortezomib–cyclophosphamide–dexamethasone
CR: Case report
CMR: Cardiac magnetic resonance
BPM: Beats per minute
AV block: Atrioventricular block
ATTR: Transthyretin amyloidosis
AL: Amyloid light-chain
